# Any detectable thyroglobulin in lymph node biopsy washouts suggests local recurrence in differentiated thyroid cancer

**DOI:** 10.1530/EC-14-0071

**Published:** 2014-09-03

**Authors:** Natalie Su-Jing Yap, Richard Maher, Diana Louise Learoyd

**Affiliations:** 1 Department of Endocrinology, Royal North Shore Hospital, St Leonards, New South Wales, Australia; 2 Department of Radiology, Royal North Shore Hospital, St Leonards, New South Wales, Australia; 3 Sydney Medical School, University of Sydney, Sydney, New South Wales, Australia

**Keywords:** thyroglobulin, lymph node, differentiated thyroid cancer, local recurrence, washout

## Abstract

The sensitivity of local recurrence detection in differentiated thyroid cancer (DTC) is increased by measuring thyroglobulin in needle washouts from lymph node fine-needle aspiration biopsies (FNA-Tg). Recent studies have proposed minimum diagnostic threshold values for FNA-Tg and have reported interference from Tg antibodies (Tg Ab), leading to low or false-negative results. The aim of this study was to assess the utility of FNA-Tg in the diagnosis of local DTC recurrence in patients referred to a single pathology service used by our tertiary teaching hospital, the first such study in an Australian cohort. Data were collected from the pathology service database for FNA-Tg over an 18-month period, and the results of 69 FNA-Tg samples from 57 patients were obtained. FNA-Tg findings were compared with cytology and histology when patients proceeded to surgery. Using the functional sensitivity as the cut-off, detectable FNA-Tg (≥0.9 μg/l) had a sensitivity of 95.7%, specificity of 50% and positive predictive value of 95.7%. Our results suggest that detectable FNA-Tg leads to histological confirmation of local nodal DTC recurrence and would support a decision to proceed to surgery. Serum Tg Ab can, however, interfere with FNA-Tg measurements. Thus, we now recommend routine use of FNA-Tg washouts in all lymph node FNA biopsies for the detection of DTC recurrence.

## Introduction

Differentiated thyroid carcinoma (DTC) is the most common endocrine malignancy, of which the majority are papillary thyroid carcinoma (PTC). While prognosis is generally favourable, up to 20% of patients develop local or regional recurrences after initial surgery (1, 2), and this occurs predominantly in the cervical lymph nodes. Ultrasonography has high sensitivity in the detection of cervical metastases but low specificity due to frequently occurring benign lymphadenopathies (3). Fine-needle aspiration cytology (FNA-C) is used to differentiate benign from malignant lymphadenopathies; however, false-negative (6–18%) (4, 5, 6) and non-diagnostic (up to 20%) (6, 7, 8, 9) results are not uncommon, particularly with cystic metastases and very small lymph nodes. It is now accepted that the sensitivity of detecting DTC in lymph node FNA-C is increased by measuring thyroglobulin in needle washouts from FNA biopsy (FNA-Tg) (4, 5, 8, 10, 11, 12, 13, 14); however, the diagnostic threshold has not been well established. Recent studies have reported interference from Tg antibodies (Tg Ab) leading to low or false-negative results (5, 9). The aim of this study was to assess the utility of FNA-Tg in the diagnosis of local DTC recurrence in patients referred to a single pathology service used by our tertiary teaching hospital, and to report the first such series in an Australian cohort.

## Materials and methods

A retrospective audit of all Tg measurements on FNA fluid was performed using the Pacific Laboratory Medicine Services (PaLMS) database over 18 months for the period of February 2012–July 2013 inclusive. The standardised methods involved were FNA biopsy (FNAB) with the contents of the biopsy needle placed onto a slide for cytology, and the needle was then washed to obtain the FNA-Tg samples. The majority of samples were obtained by washing 1 ml of normal saline through the needle into a sterile plain tube or container. In a minority of samples, there was a variation in the amount of normal saline used. The IMMULITE 2000 assay (calibrated to CRM 457 standard) was used for the measurement of serum Tg and FNA-Tg (functional sensitivity, 0.9 μg/l; analytical sensitivity, 0.2 μg/l and interassay coefficient of variation (CV), 6.9%). Tg Ab was measured using the Siemens IMMULITE 2000 (New York, NY, USA) assay (functional sensitivity, 40 kIU/l; analytical sensitivity, 20 kIU/l and CV, 9%) until October 2012, with the Abbott ARCHITECT assay (functional sensitivity, 1 kIU/l; analytical sensitivity, 1 kIU/l and CV, 4.8%; detectable defined as ≥4.4 kIU/l) used for Tg Ab analyses thereafter. All low-reading samples were diluted out to exclude hook effect. Informed consent was obtained for participation by treating clinicians. Patient demographic and clinical details were obtained through hospital medical records, as well as correspondence from treating physicians and surgeons. FNA-Tg findings were compared with histology as the gold standard when patients proceeded to surgery, as well as with FNA-C where available (the low numbers of cytology reflect one specific clinician who does not routinely perform FNA-C, although our usual practice is to perform both FNA-Tg and FNA-C at the time of FNAB).

**Figure 1:**
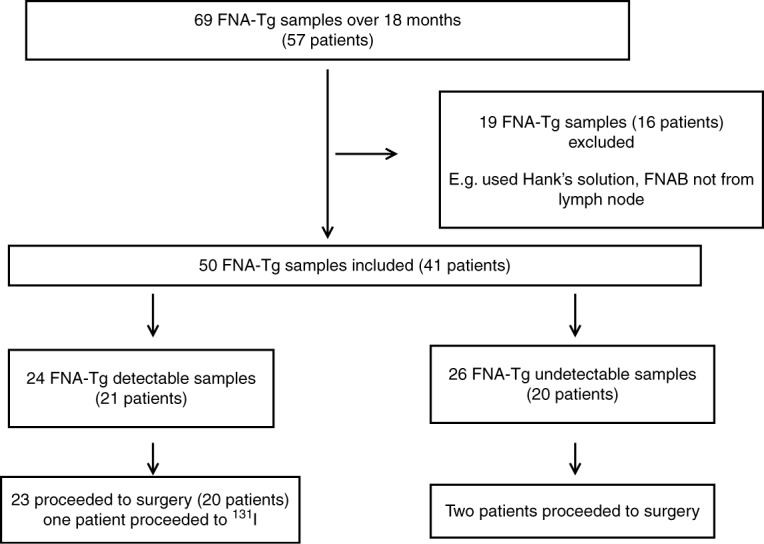
Study design flow chart. The sequential steps used in the selection and analysis of data obtained from the retrospective audit are indicated. FNA-Tg, thyroglobulin in needle washouts from fine-needle aspiration biopsy; FNAB, FNA biopsy.

The results of 69 FNA-Tg samples from 57 patients were obtained and analysed (Fig. 1). Twelve patients had more than one FNA-Tg sample taken. Nineteen FNA-Tg samples (from 16 patients) were excluded for the following reasons: samples being collected in Hank's solution, samples not collected for the purpose of DTC detection (e.g. to differentiate a thyroid nodule from a parathyroid adenoma), or inadequate information was available to include in the audit. All included FNA-Tg samples were obtained during ultrasound-guided FNAB of suspicious lymph nodes found on routine ultrasound screening or lymph nodes that were positive on PET scanning in patients with persistently positive serum Tg or Tg Ab. In the remaining 50 FNA-Tg samples (from 41 patients), 24 samples (from 21 patients) contained detectable FNA-Tg (≥0.9 μg/l) and 26 samples (from 20 patients) contained undetectable FNA-TG (<0.9 μg/l). All but one of the detectable FNA-Tg group proceeded to surgery. Only two patients in the undetectable Tg group proceeded to surgery. The characteristics of patients with detectable and undetectable FNA-Tg were compared in Table 1.

## Results

The presence of detectable serum Tg, FNA-Tg antibodies (FNA-Tg Ab) and serum Tg Ab was compared between the two groups (Table 2).

### Undetectable FNA-Tg group (<0.9 μg/l)

In the undetectable (<0.9 μg/l) FNA-Tg group, 26 samples were taken from 20 patients. 12/26 samples had negative FNA-Tg Ab in the washout fluid, and FNA-Tg Ab was not tested in 14/26 samples. Serum Tg was detectable at the time of FNAB in 5/26 samples, undetectable in 19/26 samples and not performed in 2/26 samples. Serum Tg Ab was positive at the time of FNAB in 5/26 samples, negative in 18/26 samples and not performed in 3/26 samples. The serum Tg measurements in the serum Tg Ab-positive group were all undetectable. In the patients who were serum Tg Ab-negative, serum Tg ranged from undetectable to 13.5 μg/l. FNA-C was performed in 16/26 FNABs. Of these, 13/26 were benign or reactive, 3/26 were non-diagnostic and none were malignant.

### Detectable FNA-Tg group (≥0.9 μg/l)

In the detectable (≥0.9 μg/l) FNA-Tg group, 24 samples were taken from 21 patients. FNA-Tg ranged from 0.9 to >30 000 μg/l. Interestingly, 3/24 samples had detectable Tg Ab present in the washout fluid (≥4.4 kIU/l) and FNA-Tg measured from 164.7 to >30 000 μg/l in those patients. Serum Tg Ab were positive at the time of FNAB in 5/24 samples, negative in 16/24 samples and not performed in 3/24 samples. The FNA-Tg in serum Tg Ab-positive patients ranged from 0.9 to 19 000 μg/l. FNA-Tg in serum Tg Ab-negative patients ranged from 2 to >30 000 μg/l. Serum Tg levels in the Tg Ab-positive group ranged from <0.9 to 158 μg/l and was not available in one patient. Serum Tg levels in the Tg Ab-negative group ranged from <0.9 to 388 μg/l. FNA-C was performed at the time of FNAB in 16/24 samples, and 13/24 were malignant, 1/24 was reactive and 2/24 were non-diagnostic. FNA-C was not performed in eight samples for reasons previously stated.

### Patients who proceeded to surgery

Histology was used as the gold standard to compare the performance of FNA-Tg (Table 3) and FNA-C (Table 4) for those who proceeded to surgery. All but one patient in the detectable FNA-Tg group proceeded to surgery, and the remaining patient proceeded to ^131^I ablation. Histology was positive in all 20 patients who proceeded to surgery, except one patient. This ‘false positive’ occurred in one patient with detectable FNA-Tg (22 μg/l), undetectable serum Tg, positive serum Tg Ab and negative histology; however, this patient had thyroid rest cells confirmed histologically in an ipsilateral neck region. Only two patients with undetectable FNA-Tg proceeded to surgery. The first patient had strongly positive serum Tg Ab (340 kIU/l) with an undetectable FNA-Tg despite clearly pathological features on ultrasound. She proceeded to surgery based on the suspicious ultrasound features and repeat FNA-Tg of the lymph node intra-operatively reached just detectable limits at 0.9 μg/l and histology confirmed PTC. The second patient had a suspicious lymph node biopsied prior to surgery as part of her original diagnosis of DTC and so had an intact thyroid gland at the time of biopsy. She, therefore, proceeded to total thyroidectomy and neck dissection despite the undetectable FNA-Tg in this particular lymph node and histology was negative. Using 0.9 μg/l as the cut-off, detectable FNA-Tg has a sensitivity of 95.7%, specificity of 50% and positive predictive value (PPV) of 95.7%. Fifteen patients with both FNA-C and histology showed that malignant FNA-C had a sensitivity of 81.3%, specificity of 100% and PPV of 100%.

### Patients with intact thyroid glands at the time of nodal FNA

Six patients had FNABs performed for suspicious lymph nodes found at the time of initial thyroid nodule biopsy and were therefore sampled prior to thyroidectomy for PTC. Four patients had detectable FNA-Tg and two had undetectable FNA-Tg. In the four patients with detectable FNA-Tg, FNA-Tg ranged from 1 to >30 000 μg/l. All four with detectable FNA-Tg proceeded to surgery and had positive histology.

### Performance of FNA-Tg vs cytology

A total of 30 patients had both FNA-Tg and FNA-C performed. FNA-C was diagnostic (i.e. benign or malignant) vs insufficient or non-diagnostic in 25 of these patients and confirmatory histology was available for 14 of these patients. FNA-Tg and FNA-C were concordant in all samples except one patient who had a detectable FNA-Tg, and positive histology, but cytology demonstrated a polymorphous lymphoid population, consistent with a reactive lymph node. FNA-C was non-diagnostic in five of the 30 patients. Of these, two patients had detectable FNA-Tg and proceeded to surgery, with malignancy confirmed on histology. The combination of a detectable FNA-Tg and/or a malignant FNA-C resulted in sensitivity, specificity and PPV of 100% for all 15 patients with both procedures performed.

## Discussion

The measurement of Tg in the needle washout after aspiration was first proposed in 1992 by Pacini *et al*. (15) for the detection of lymph node metastases in DTC patients. Since then, it has been well demonstrated that FNA-Tg is more sensitive than FNA-C in diagnosing local nodal recurrence in DTC. Reported sensitivities for FNA-Tg vary depending on the cut-offs used and range from 81.4 to 100% vs 55 to 85% (1, 4, 5, 8, 9, 10, 15, 16, 17) for FNA-C. The current American Thyroid Association guidelines recommend biopsy of suspicious lymph nodes for FNA-C and FNA-Tg where a positive result would change management (18). The current European Thyroid Association guidelines (14) concur, taking into account the stage and histology of the disease, size and location of the lymph nodes and the serum Tg level. In the absence of clear international consensus, our institution utilises various guidelines on a case-by-case basis.

Our data suggest that there can be a wide spectrum of results obtained from FNA-Tg and two cases are particularly illustrative. A patient who had undergone initial surgery and radioactive iodine ablation had positive FDG–PET uptake in the right level II/III lymph nodes in the context of high serum Tg, negative Tg Ab and negative ultrasound-guided FNA-C on two occasions. A PET with ultrasound-guided biopsy yielded FNA-Tg 11 600 μg/l and non-diagnostic FNA-C with PTC confirmed on histology. Another patient who underwent thyroidectomy and radioactive iodine ablation for a sclerosing-variant PTC with vascular invasion had persistent elevation of serum Tg Ab with an undetectable serum Tg, and a surveillance ultrasound showed a lesion near the left thyroid bed. FNA-C confirmed nodal PTC and FNA-Tg was low positive at 1.9 μg/l with PTC confirmed on histology. While the sensitivity of FNA-Tg is very high, we nevertheless recommend performing FNA-Tg in conjunction with FNA cytology, particularly in the presence of serum Tg Ab that can result in FNA-Tg false negatives. Our findings for the 15 patients who had both FNA-Tg and FNA-C performed showed a sensitivity, specificity and PPV of 100% for those who had one or both tests positive.

A number of potential pitfalls exist in preparing the sample for FNA-Tg measurement and assessing appropriate cut-off levels. There is a disagreement about whether the washout solution should be Tg-free serum (the standard ‘0’ provided by the Tg kit company) (15) or normal saline (4, 8, 16, 18). Other problems include samples collected in lithium–heparin tubes (19) and the hook effect (where very high Tg concentrations result in excess antigen saturating the binding capacity of the Tg capture antibody) can also lead to false negatives. The use of the international reference standard (CRM 457) has significantly reduced inter-method variability (20). Cut-off levels used in published studies vary significantly (21), from the functional sensitivity of the assay to the mean±s.d. of patients with negative cytology, to ‘best fit’ using the area under the receiver operating characteristic curve to limit false negatives. A recent review by Torres *et al*. (21) recommends a cut-off of >10 ng/ml (>10 μg/l) to identify those appropriate for neck dissection. A recent very large retrospective cohort study of 528 cases using an ultra-sensitive Tg assay validated 1.0 ng/ml as a cut-off value for diagnosing PTC nodal metastases, and this cut-off has been supported by a recent meta-analysis (22). The presence of serum Tg Ab or Tg Ab in washout fluid (thought to be due to blood contamination or Tg Ab synthesis within the node) resulted in lower FNA-Tg concentrations in our study and in others (5, 9). It has been reported that stimulation with recombinant human thyrotropin can help overcome this issue (23); however, this expensive and complicated approach is unlikely to be incorporated into routine clinical practice. Anecdotal experience with the ultra-sensitive Tg assay used in our institution (ELISA; functional sensitivity, 0.05 μg/l and analytical sensitivity, 0.02 μg/l) indicates that it may be less affected by Tg Ab levels; however, further data are required to confirm this.

Few authors have examined the difference between athyrotic patients and DTC patients with intact thyroids (4, 5, 10). The risk of blood contamination in individuals with an intact thyroid and inadvertent passage of biopsy needles through the thyroid gland during biopsy have led some to propose that a higher cut-off is required for these patients (5). In our study, the presence of one patient with FNA-Tg of 1 μg/l which proved to be malignant on histology would argue against using a higher cut-off in patients awaiting thyroidectomy.

In summary, all patients with FNA-Tg levels at or above that of the functional sensitivity of the assay (≥0.9 μg/l) had histologically confirmed nodal PTC recurrence except one patient who had thyroid rest cells confirmed histologically in the ipsilateral neck region and another who proceeded to ^131^I therapy rather than surgery, and thus histology was not available. There were no patients with histologically confirmed nodal recurrence who had undetectable FNA-Tg except one patient who had positive serum Tg Ab.

The strengths of our study are the consistency of methods and the correlation of FNA-Tg with the gold standard of histology. Our study confirms the high sensitivity of nodal Tg measurement and confirms that it should be performed in all patients undergoing nodal FNA to detect recurrence of DTC. A standardised protocol should be adopted for FNA-Tg sample collection and interpretation to allow more accurate comparison of data from different centres.

## Figures and Tables

**Table 1:** Characteristics of detectable and undetectable FNA-Tg groups.

	**FNA-Tg detectable**	**FNA-Tg undetectable**
Samples (*n*=24), patients (*n*=21)	Samples (*n*=26), patients (*n*=20)
Mean age (years)^a^	48±17	49±11
Female^a^	17/21	12/20
New diagnosis of DTC	5/24	1/26
Previous DTC	19/24	25/26
Histology subtype		
Papillary thyroid carcinoma	16/24	23/26
Follicular variant	0/24	3/26
Sclerosing variant	1/24	0/26
Tall cell variant	2/24	0/26
Hürthle cell variant	1/24	0/26
Mixed type	3/24	0/26
Hürthle cell carcinoma	1/24	0/26
AJCC/UICC TNM staging at diagnosis		
I	12/24	14/26
II	1/24	2/26
III	2/24	6/26
IVa	7/24	2/26
IVb	1/24	0/26
IVc	0/24	1/26
Not available	1/24	1/26

FNA-Tg, thyroglobulin in needle washouts from fine-needle aspiration biopsy; DTC, differentiated thyroid carcinoma; AJCC/UICC TNM, American Joint Committee on Cancer/International Union against Cancer TNM Classification System for Differentiated Thyroid Carcinoma.

a Numbers are based on patient numbers not sample numbers.

**Table 2:** FNA-Tg Ab, serum Tg, serum Tg Ab and cytology results for detectable and undetectable FNA-Tg groups.

	**FNA-Tg detectable**	**FNA-Tg undetectable**
Samples (*n*=24)	Samples (*n*=26)
FNA-Tg Ab		
Positive	3/24	0/26
Negative	9/24	12/26
Not done	12/24	14/26
Serum Tg		
Detectable	13/24	5/26
Undetectable	7/24	19/26
Not done	4/24	2/26
Serum Tg Ab		
Detectable	5/24	5/26
Undetectable	16/24	18/26
Not done	3/24	3/26
Cytology (FNA-C)		
Malignant	13/24	0/26
Non-malignant (benign/reactive)	1/24	13/26
Non-diagnostic	2/24	3/26
Not done	8/24	10/26

FNA-Tg Ab, thyroglobulin antibodies in needle washouts from fine-needle aspiration biopsy; Tg, thyroglobulin; serum Tg Ab, serum Tg antibodies; FNA-C, FNA biopsy cytology.

**Table 3:** Lymph node histology vs FNA-Tg.

	**Histology malignant**	**Histology benign**	**Total**
Detectable FNA-Tg (≥0.9 μg/l)	22	1	23
Undetectable FNA-Tg (<0.9 μg/l)	1	1	2
Total	23	2	25

FNA-Tg, thyroglobulin in needle washouts from fine-needle aspiration biopsy.

**Table 4:** Lymph node histology vs FNA-C.

	**Histology malignant**	**Histology benign**	**Total**
Cytology malignant	13	0	13
Cytology benign/non-diagnostic	3	1	4
Total	16	1	17

FNA-C, fine-needle aspirate biopsy cytology.
